# Association of neutrophil to lymphocyte ratio with all-cause and cardiovascular mortality among individuals with kidney stone disease: result from NHANES, 2007-2018

**DOI:** 10.3389/fendo.2025.1537403

**Published:** 2025-03-14

**Authors:** Qihui Chu, Bin Wu, Zhaofu Zhang

**Affiliations:** ^1^ Department of Clinical Laboratory, the First People’s Hospital of Yuhang District, Hangzhou, China; ^2^ Department of Infectious Diseases, The Seventh Affiliated Hospital, Sun Yat-sen University, Shenzhen, China

**Keywords:** NHANES, kidney stone, inflammation, neutrophil to lymphocyte ratio, mortality

## Abstract

**Background:**

The objective of this study is to investigate the relationship between the neutrophil-to-lymphocyte ratio (NLR) and all-cause as well as cause-specific mortality among patients with kidney stones, and to evaluate the capability of NLR as a predictor of mortality.

**Methods:**

This study included 2,995 patients with kidney stones from the NHANES database during the period from 2007 to 2018, and subsequently linked this data with the National Death Index. The relationship between NLR and mortality was analyzed using the Cox proportional hazards model and Kaplan-Meier survival curves. Additionally, restricted cubic spline (RCS) curves were employed to explore the dose-response relationship between NLR and mortality, while time-dependent ROC curves were utilized to assess the predictive capability of NLR for mortality. Finally, the mediating effect of estimated glomerular filtration rate (eGFR) on the relationship between NLR and mortality was also analyzed.

**Results:**

This study ultimately included 2,995 patients with kidney stones, with a median follow-up period of 74 months. A total of 395 deaths were recorded, of which 87 were attributed to cardiovascular diseases. An NLR cut-off of 3.62 was identified as significantly associated with survival outcomes using the ‘maxstat’ package and the principle of maximum rank statistics. The restricted cubic spline plot indicates a non-linear relationship between NLR and both all-cause mortality and cardiovascular mortality. After adjusting for relevant covariates, the Cox regression analysis demonstrated that, in comparison to the lower NLR group, the higher NLR group exhibited a 1.05-fold (HR 2.05, 95% CI 1.51-2.78, *P* < 0.001) increased risk of all-cause mortality and a 1.99-fold (HR 2.99, 95% CI 1.89-4.72, *P* < 0.001) increased risk of cardiovascular mortality. Furthermore, eGFR exhibited a significant mediating effect on the relationship between NLR and mortality.

**Conclusion:**

This study found that patients with kidney stones exhibiting a high NLR have a significantly increased risk of mortality in the U.S. population. Therefore, monitoring NLR may be important for the prognosis of patients with kidney stones.

## 1 Introduction

Kidney stones represent a notable global health issue, with increasing incidence rates documented across diverse geographic regions. Previous research has estimated that the prevalence of kidney stones in the U.S. population is approximately 8.8%, indicating that roughly one in every eleven individuals is affected by this condition (1). The growing incidence is attributed to various factors, including dietary habits, increased body mass index (BMI), and climate changes affecting fluid intake (2). The healthcare burden associated with kidney stones is considerable. In addition to the acute pain and morbidity experienced by patients, the economic impact on healthcare systems is significant. It is estimated that the direct medical costs for kidney stone management in the United States alone exceed $10 billion annually (3).

Inflammation is closely associated with kidney stones. A previous study indicated that the biomarkers C-reactive protein and erythrocyte sedimentation rate are closely associated with kidney stones (4). the neutrophil-to-lymphocyte ratio (NLR) is a novel inflammatory marker that combines neutrophil and lymphocyte counts from routine blood tests and is associated with an increased risk of kidney stone formation (5, 6). The NLR also has high efficacy in predicting mortality, both in the general population and in some specific populations (7–10). However, there is currently no study available regarding the association between NLR and the risk of mortality from kidney stones.

To date, there is a paucity of study examining the relationship between NLR and the prognosis of individuals with kidney stones. This study draws upon data from the National Health and Nutrition Examination Survey (NHANES) with the aim of comprehensively analyzing the prognostic value of NLR in patients with kidney stones.

## 2 Materials and methods

### 2.1 Data source

This study primarily utilized data from 7 consecutive cycles of the NHANES database spanning from 2007 to 2018. The database uses random, stratified, and weighted sampling methods and contains physical examination, dietary, and other relevant data from U.S. residents (11). NHANES is conducted by the National Center for Health Statistics (NCHS) within the Centers for Disease Control and Prevention (CDC), and informed consent was obtained from all participants.

### 2.2 Study populations

**Figure 1:**
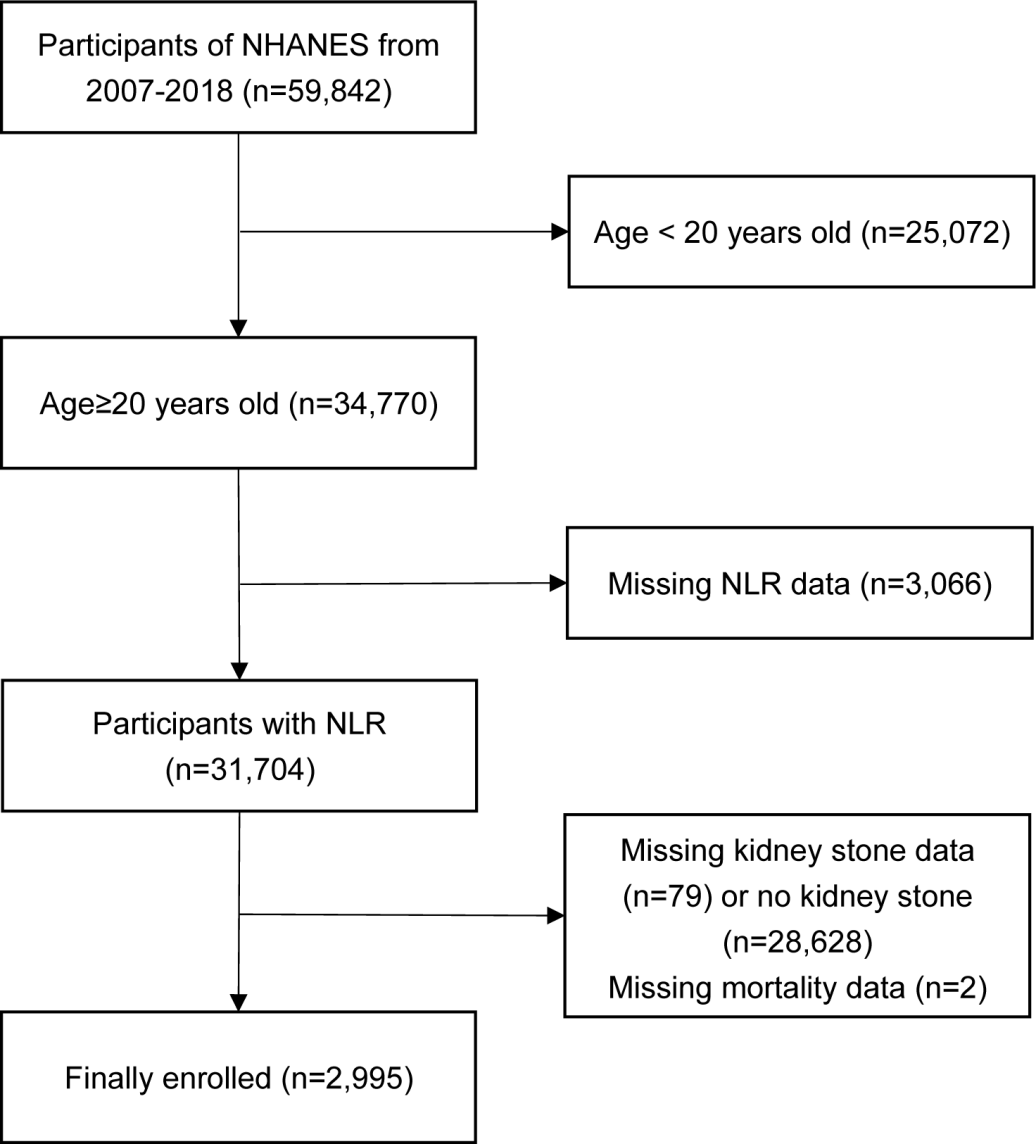
Flow-chart of the participants’ selection. NHANES, National Health and Nutrition Examination Survey; NLR, neutrophil-to-lymphocyte ratio.

This prospective cohort study selected participants from the continuous NHANES population conducted between 2005 and 2018. The diagnosis of kidney stones was based on questionnaire data, specifically the question, “Ever had kidney stones?” Those who responded “yes” were confirmed to have a history of kidney stones (12). This study included a total of 59,842 participants, excluding those under the age of 20 (25,072 individuals). Subsequently, we excluded participants with missing neutrophil and lymphocyte counts in the complete blood count, those lacking kidney stone questionnaire data, those without a history of kidney stones, and those missing mortality data. Ultimately, the number of kidney stone participants included in the statistical analysis was 2,995 (**Figure 1**).

### 2.3 Measurement of NLR

The Beckman Coulter MAXM instrument utilized in the Mobile Examination Center (MEC) performs complete blood counts on blood specimens, generating blood cell distribution data for all participants. The calculation of NLR is based on the ratio of neutrophils to lymphocytes obtained from routine complete blood counts (13).

### 2.4 Determination of mortality and follow-up time

The NHANES data were combined with records from the National Death Index (NDI) to determine the mortality status of participants, with follow-up time ending on December 31, 2019. The International Classification of Diseases, Tenth Revision (ICD-10), was employed to delineate potential causes of death. The National Center for Health Statistics (NCHS) specifically categorizes cardiovascular mortality as deaths attributable to heart disease, which are defined by the ICD-10 codes I00-I09, I11, I13, and I20-I51 (14).

### 2.5 Covariates

The covariates incorporated in the analysis of this study included age, sex, race, income level, education level, physical activity, alcohol consumption, smoking status, body mass index (BMI), estimated glomerular filtration rate (eGFR), total cholesterol, high-density lipoprotein cholesterol, diabetes, hypertension, cardiovascular disease, a history of cancer, drugs, HEI-2015, and health insurance. Race was classified into the following categories: Mexican American, Other Hispanic, Non-Hispanic White, Non-Hispanic Black, and Other Race, which includes Multi-Racial individuals. Income level was represented by the poverty income ratio (PIR), categorized as low, medium, and high (15). Physical activity was categorized as never, moderate, and vigorous. Smoking status was classified into three distinct categories: never smoker, former smoker, and current smoker. Obesity was defined as a BMI of ≥ 30 kg/m². Diabetes is diagnosed based on the patient’s medical history, the use of oral hypoglycemic medications or insulin therapy, a fasting blood glucose level of ≥ 126 mg/dL, or a hemoglobin A1c level of ≥ 6.5% (16). The diagnosis of hypertension was determined based on self-reported medical history, the utilization of antihypertensive medications, or a recorded blood pressure measurement of ≥ 140/90 mmHg (17). Cardiovascular disease and cancer are diagnosed based on self-reported medical history from the questionnaire data. The glomerular filtration rate was assessed using the Chronic Kidney Disease Epidemiology Collaboration (CKD-EPI) formula (18). Drugs include common prescription drugs for the treatment of diabetes, hypertension, and hyperlipidemia. According to the Dietary Guidelines for Americans, the HEI-2015 score is calculated based on 13 food components (19).

### 2.6 Statistical analysis

In alignment with the design characteristics of the National Health and Nutrition Examination Survey (NHANES), this study rigorously accounted for the intricate features of the study design, using a weight of (1/6 * WTMEC2YR) (20). Continuous variables were presented as weighted means and standard errors, while categorical variables were represented as weighted counts and percentages. The differences between two groups of continuous variables and categorical variables were assessed using Student’s t-test and chi-square test, respectively.

We employed the ‘maxstat’ package (https://CRAN.R-project.org/package=maxstat) to determine the optimal cutoff value for NLR impacting survival outcomes, utilizing the methodology of maximum rank statistics. Subsequently, participants were stratified into a lower NLR group and a higher NLR group based on this established cutoff value (21–23). The “rms” package was used to plot restricted cubic splines (RCS) to provide a more intuitive representation of the nonlinear relationship between NLR and both all-cause and cardiovascular mortality in participants with kidney stones. The “survival” package was used to conduct Cox proportional hazards models to examine the relationship between NLR and mortality. Model 1 was a crude model; Model 2 incorporated age, sex, and race; while Model 3 further adjusted Model 2 to account for additional factors including income level, education level, physical activity, alcohol consumption, smoking status, body mass index (BMI), eGFR, total cholesterol, high-density lipoprotein cholesterol, diabetes, hypertension, cardiovascular disease, and cancer. In survival analysis, due to the limited number of CVD-specific deaths among patients with kidney stones, Least Absolute Shrinkage and Selection Operator regression (LASSO) was used to select important covariates in order to simplify the model. In addition, we also used the “survival” package and the “survminer” package to plot Kaplan-Meier survival curves and perform the log-rank test, in order to more intuitively reflect the differences in mortality between the lower NLR group and the higher NLR group. Analyses were also stratified by age, sex, race, obesity, smoking status, education level, diabetes and hypertension, and *P*-values for interaction between NLR and different subgroups were calculated. The “timeROC” package was utilized to conduct time-dependent ROC curve analysis to compare the effectiveness of NLR in predicting mortality across different follow-up durations (24). In addition, the “riskRegression” package was used to plot continuous time-dependent ROC curves to compare the predictive performance of NLR and other inflammatory indicators. In the mediation analysis, the “mediation” package was used to estimate the mediation effect through the Bootstrap method with 5000 repeated simulations (25, 26). All data analyses were conducted using R Statistical Software version 4.4.1 (http://www.r-project.org). A two-tailed *p*-value of less than 0.05 was deemed statistically significant.

## 3 Results

### 3.1 Demographic and clinical characteristics of the study population

**Figure 2:**
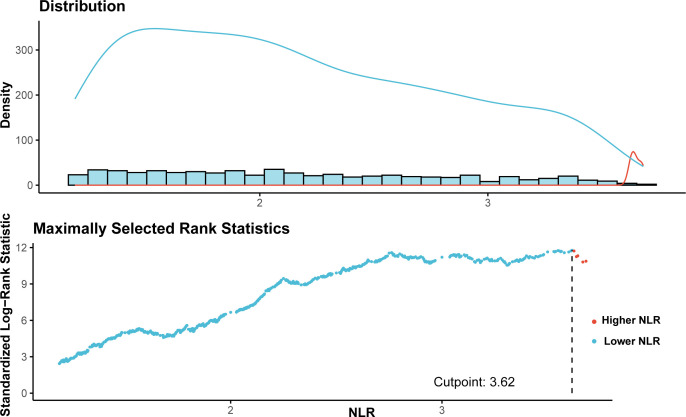
NLR distribution and optimal cutpoint analysis. NLR, neutrophil-to-lymphocyte ratio.

A total of 2,995 kidney stone participants were recruited in this study, representing 20,681,725 kidney stone patients in the United States. Utilizing the ‘maxstat’ package and employing the principle of maximum rank statistics, an NLR cutoff value of 3.62 was identified as significantly associated with survival outcomes. Accordingly, based on the NLR cutoff value of 3.62, participants were categorized into a higher group (n = 322) and a lower group (n = 2,673) (**Figure 2**). In comparison to participants in the low NLR group, individuals in the high NLR group were older, exhibited a higher proportion of males, and demonstrated increased rates of obesity, as well as a greater prevalence of diabetes, hypertension, cardiovascular disease (CVD), and cancer. Furthermore, participants in the high NLR group exhibited lower lymphocyte counts, total cholesterol (TC) levels, and eGFR, while neutrophil counts were elevated. Additional baseline characteristics of the participants are presented in **Table 1**.

**Table 1:** Basic characteristics of participates with kidney stone based on NLR category.

Characteristics	Overall	Lower NLR	Higher NLR	P value
n	2,995	2,673	322	
N	20,681,725	18,493,937	2,187,788	
Age,years	53.52 (0.35)	52.77 (0.36)	59.80 (1.13)	<0.001
Sex,%				<0.001
Male	11,247,408 (54.38)	9,817,518 (53.09)	1,429,890 (65.36)	
Female	9,434,317 (45.62)	8,676,419 (46.91)	757,898 (34.64)	
Race/ethnicity, %				0.059
Mexican American	1,277,548 (6.18)	1,163,253 (6.29)	114,295 (5.22)	
Other Hispanic	1,066,761 (5.16)	965,219 (5.22)	101,542 (4.64)	
Non-Hispanic White	15,867,133 (76.72)	14,056,436 (76.01)	1,810,697 (82.76)	
Non-Hispanic Black	1,202,096 (5.81)	1,138,052 (6.15)	64,044 (2.93)	
Other race	1,268,187 (6.13)	1,170,977 (6.33)	97,210 (4.44)	
Poverty-income ratio,%				0.015
low	2,513,169 (12.15)	2,313,078 (12.51)	200,091 (9.15)	
moderate	8,595,359 (41.56)	7,532,230 (40.73)	1,063,129 (48.59)	
high	9,573,197 (46.29)	8,648,629 (46.76)	924,568 (42.26)	
Educational level,%				0.062
< high school	3,277,282 (15.85)	2,847,302 (15.40)	429,980 (19.65)	
≥ high school	17,404,443 (84.15)	15,646,635 (84.60)	1,757,808 (80.35)	
Physical activity,%				0.004
Never	11,110,808 (53.72)	9,852,649 (53.28)	1,258,159 (57.51)	
Moderate	4,603,967 (22.26)	4,039,185 (21.84)	564,782 (25.82)	
Vigorous	4,966,950 (24.02)	4,602,103 (24.88)	364,847 (16.68)	
Alcohol drinker,%				0.944
No	8,681,275 (41.98)	7,767,807 (42.00)	913,468 (41.75)	
Yes	12,000,450 (58.02)	10,726,130 (58.00)	1,274,320 (58.25)	
Smoking status,%				0.111
Never	10,441,881 (50.49)	9,455,552 (51.13)	986,329 (45.08)	
Former	6,179,037 (29.88)	5,434,542 (29.39)	744,495 (34.03)	
Current	4,060,807 (19.63)	3,603,843 (19.49)	456,964 (20.89)	
BMI, kg/m^2^	30.63 (0.17)	30.66 (0.18)	30.32 (0.58)	0.401
BMI status (kg/m2 ), %				0.029
<30	10,650,273 (51.50)	9,403,201 (50.84)	1,247,072 (57.00)	
≥30	10,031,452 (48.50)	9,090,736 (49.16)	940,716 (43.00)	
Total cholesterol (mmol/L)	4.96 (0.28)	5.00 (0.03)	4.66 (0.07)	<0.001
High-density lipoprotein (mmol/L)	1.29 (0.01)	1.29 (0.01)	1.28 (0.03)	0.614
eGFR, mL/min/1.73 m^2^	87.77 (0.49)	88.65 (0.52)	80.34 (1.51)	<0.001
Diabetes mellitus, %	5,172,603 (25.01)	4,435,025 (23.98)	737,578 (33.71)	<0.001
Hypertension, %	10,956,069 (52.97)	9,642,448 (52.14)	1,313,621 (60.04)	0.006
CVD,%	3,292,341 (15.92)	2,799,901 (15.14)	492,440 (22.51)	<0.001
Cancer,%	3,279,665 (0.92)	2,675,481 (0.95)	604,184 (3.28)	<0.001
Lymphocyte, × 109/L	2.11 (0.02)	2.19 (0.02)	1.41 (0.04)	<0.001
Neutrophil, × 109/L	4.52 (0.05)	4.25 (0.04)	6.73 (0.24)	<0.001
HEI-2015	49.16 (0.35)	49.19 (0.36)	48.96 (0.97)	0.821
Anti-diabetic drugs				<0.001
No	17,205,127 (83.19)	15,536,756 (84.01)	1,668,371 (76.25)	
Yes	3,476,598 (16.81)	2,957,181 (15.99)	519,417 (23.75)	
Anti-hypertensive drugs				<0.001
No	11,594,175 (56.06)	10,704,291 (57.88)	889,884 (40.67)	
Yes	9,087,550 (43.94)	7,789,646 (42.12)	1,297,904 (59.33)	
Anti-hyperlipidemic drugs				<0.001
No	14,824,660 (71.68)	13,548,658 (73.26)	1,276,002 (58.32)	
Yes	5,857,065 (28.32)	4,945,279 (26.74)	911,786 (41.68)	
Health insurance				0.001
No	2,756,874 (13.33)	2,515,025 (13.61)	241,849 (11.06)	
Yes	18,104,851 (86.67)	15,978,912 (86.39)	1,945,939 (88.94)	

Data were presented as weighted mean (standard error) for continuous variables and weighted number of individuals (weighted percentage) for categorical variables.

NLR, neutrophil-to-lymphocyte ratio; eGFR, estimated glomerular filtration rate; BMI, body mass index; CVD, cardiovascular disease; HEI-2015, healthy eating index-2015.

### 3.2 The association between NLR and all cause mortality

**Figure 3:**
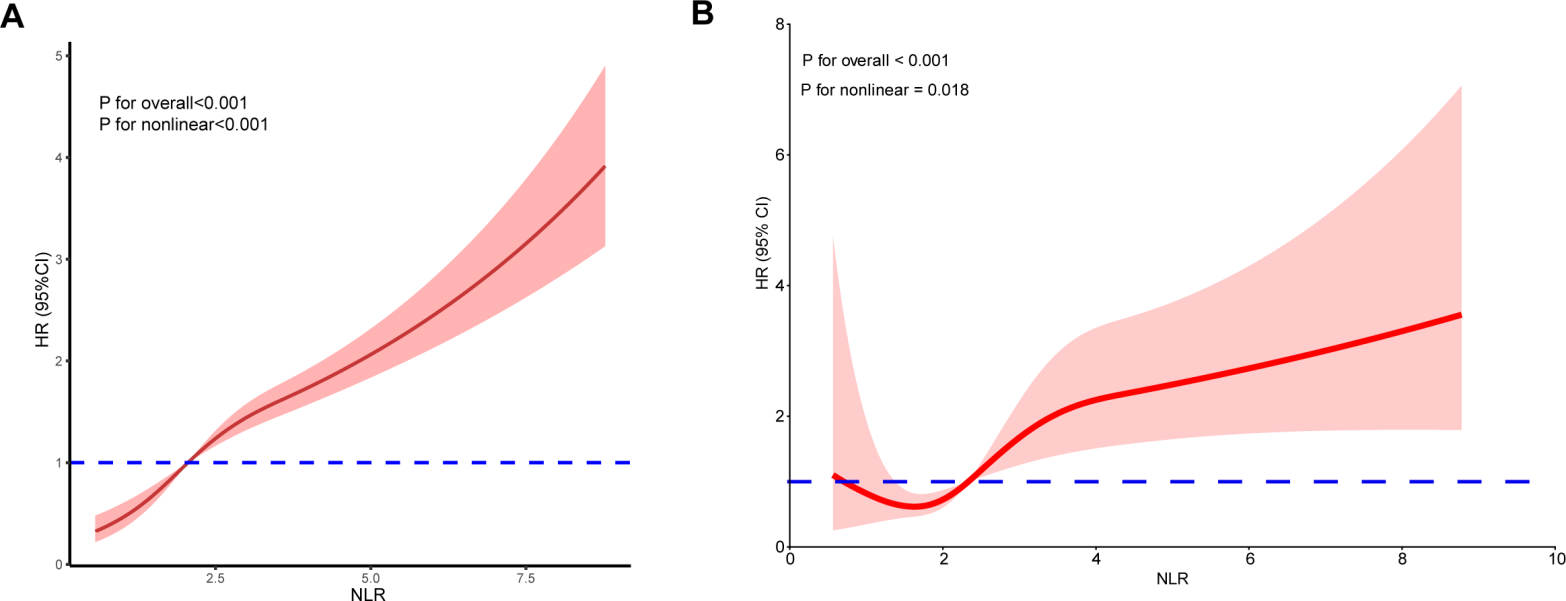
The relationship between NLR and all-cause mortality **(A)** as well as cardiovascular mortality **(B)** among patients with kidney stones is illustrated by the restricted cubic spline (RCS).

During a median follow-up period of 74 months (interquartile range [IQR], 41–115 months), a total of 395 deaths were recorded, of which 87 were attributable to cardiovascular diseases. The restricted cubic spline (RCS) curve demonstrated a nonlinear relationship between NLR and all-cause mortality, with *P*-values for nonlinear detection being less than 0.001 (**Figure 3A**). In the unadjusted model, our analysis revealed that the risk of all-cause mortality increased with higher NLR values (Model 1, HR 1.24, 95% CI 1.18–1.29, *P* < 0.001). Following adjustment for all covariates, each 1-unit increase in NLR was associated with a 17% increase in mortality risk (Model 3, HR 1.17, 95% CI 1.11–1.23, *P* < 0.001).

**Figure 4:**
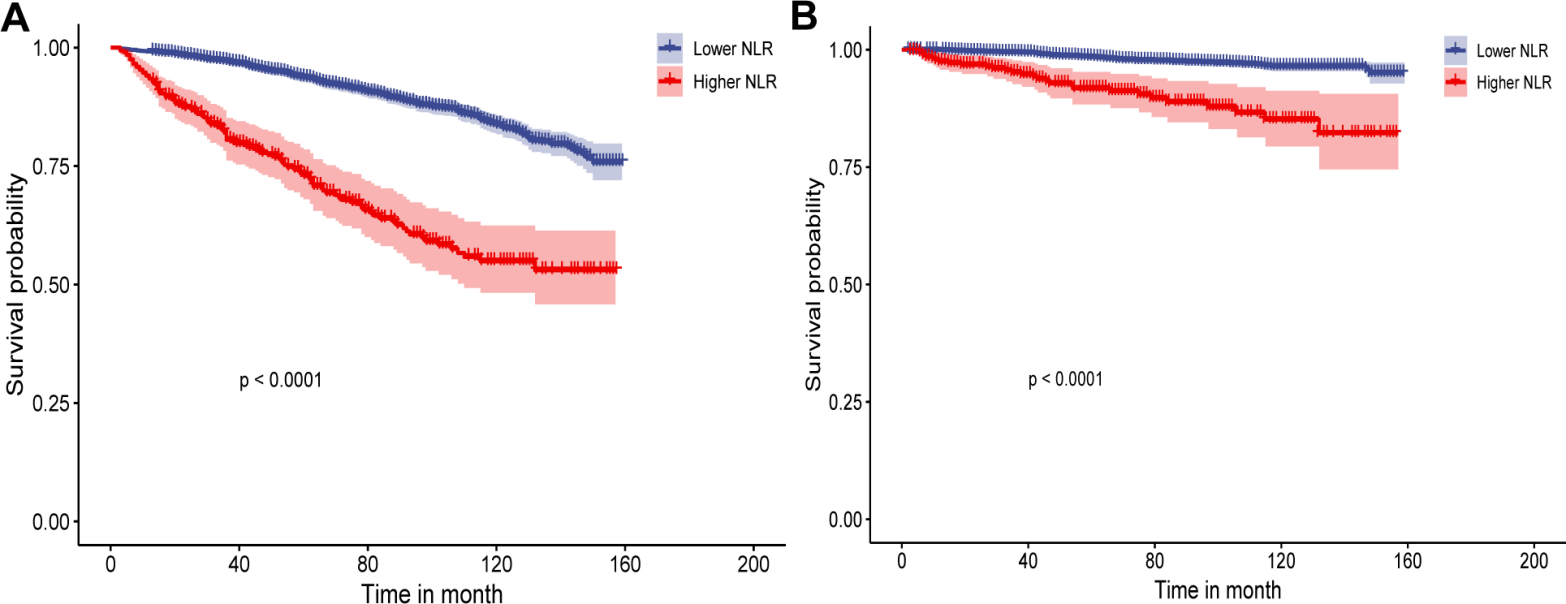
Kaplan-Meier survival curves by NLR levels all-cause **(A)** and cardiovascular disease mortality **(B)**. NLR, neutrophil-to-lymphocyte ratio.

The Kaplan-Meier survival curve indicated a significant difference in survival rates between the two groups, with the survival rate in the high NLR group surpassing that of the low NLR group (*P* < 0.001) (**Figure 4A**). Cox regression analysis, treating NLR as a binary variable, revealed that the all-cause mortality rate was significantly higher in the higher NLR group compared to the lower NLR group in all three models: Model 1 (HR 3.20, 95% CI 2.25-4.00, *P* < 0.001), Model 2 (HR 1.98, 95% CI 1.46-2.72, *P* < 0.001), and Model 3 (HR 2.05, 95% CI 1.51-2.78, *P* < 0.001) (**Table 2**).

**Table 2:** The relationships between NLR and mortality in participants with kidney stone.

Characteristics	Model 1 HR (95% CI)	P value	Model 2 HR (95% CI)	P value	Model 3 HR (95% CI)	P value
All cause mortality						
NLR	1.24 (1.18,1.29)	<0.001	1.14 (1.08,1.21)	<0.001	1.17 (1.11,1.23)	<0.001
NLR category						
Lower NLR	Ref		Ref		Ref	
Higher NLR	3.20 (2.35,4.00)	<0.001	1.98 (1.46,2.72)	<0.001	2.05 (1.51,2.78)	<0.001
Cardiovascular mortality						
NLR	1.28 (1.20,1.35)	<0.001	1.17 (1.10,1.25)	<0.001	1.19 (1.12,1.29)	<0.001
NLR category						
Lower NLR	Ref		Ref		Ref	
Higher NLR	5.91 (3.38,10.29)	<0.001	3.18 (2.01,5.01)	<0.001	2.99 (1.89,4.72)	<0.001

For All-caused-mortality.

Model 1, unadjusted; Model 2, adjusted for age, sex, race; Model 3, adjusted for age, sex, race, poverty income ratio, education level, smoking status, physical activity, body mass index, total cholesterol, high-density lipoprotein, estimated glomerular filtration rate, diabetes, hypertension, cardiovascular disease, and cancer.

For CVD-caused-mortality.

Model 1, unadjusted; Model 2, adjusted for age; Model 3, adjusted for age, estimated glomerular filtration rate, diabetes, cardiovascular disease.

**Figure 5:**
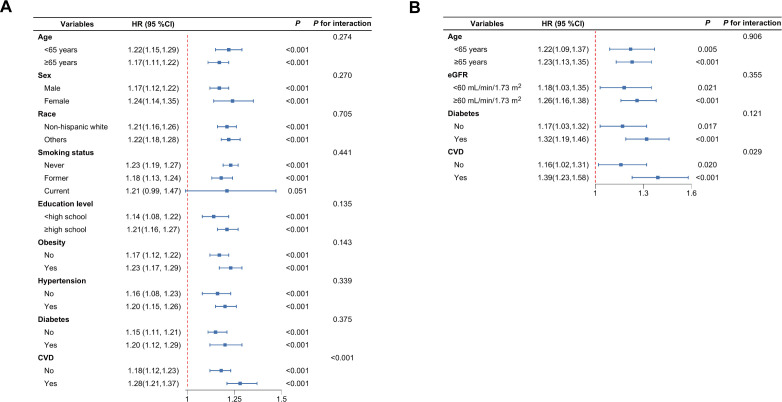
Forest plot of hazard ratios for all-cause **(A)** and cardiovascular disease mortality **(B)**. HR, Hazard ratio; CI, Confdence interval.

The stratified analyses, conducted on the basis of age, sex, race, education level, smoking status, obesity, diabetes, hypertension, and CVD, revealed that the interaction p-values for each subgroup were all higher than 0.05. This finding indicates a high degree of consistency in the results across these stratified groups (**Figure 5A**).

### 3.3 The association between NLR and cardiovascular mortality

**Figure 6:**
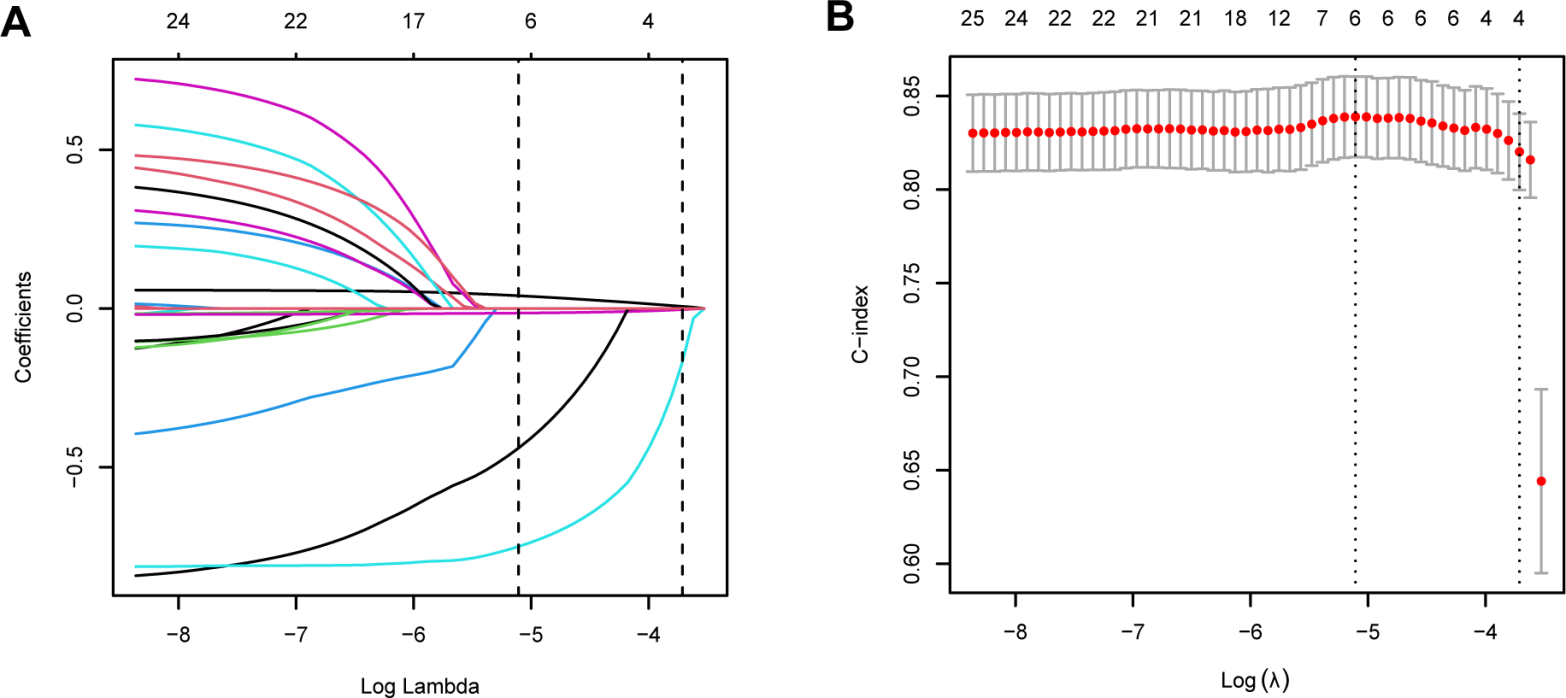
LASSO regression of 18 covariates associated with CVD mortality. **(A)** The screening path corresponds to 15 covariates that contribute to CVD mortality; **(B)** The association between the log-transformed λ and Partial Likelihood Deviance for CVD mortality. The red dashed line and its error bars represent the average Partial Likelihood Deviance value and the corresponding 95% CI.

The RCS curve indicated a nonlinear relationship between NLR and cardiovascular mortality, with P-values for nonlinear detection being less than 0.001 (**Figure 3B**). Considering that there were only 87 cases of cardiovascular deaths, we referred to previous literature and used LASSO regression to select 4 important covariates from all covariates related to all-cause mortality (27) (**Figures 6A, B**). These covariates were age, eGFR, diabetes, and CVD. Model 1 adjusts for covariates, Model 2 adjusts for age, and Model 3 adjusts for these 4 covariates. In the unadjusted model, a significant correlation was observed between higher NLR and an elevated risk of cardiovascular mortality (Model 1, HR 1.28, 95% CI 1.20–1.35, *P* < 0.001). After adjusting for all covariates, Model 3 revealed a significant association between a 1-unit increase in NLR and a 19% elevation in mortality risk (HR 1.19, 95% CI 1.12–1.29, *P* < 0.001).

The Kaplan-Meier survival curve revealed a significant difference between the two NLR groups, with the higher NLR group exhibiting a superior survival rate (*P* < 0.001) (**Figure 4B**). The results of the Cox regression analysis indicated a significant difference in all-cause mortality risk between the two groups across all three models, with the higher NLR group exhibiting a greater risk of mortality: Model 1 (HR 5.91, 95% CI 3.38-10.29, *P* < 0.001), Model 2 (HR 3.18, 95% CI 2.01-5.01, *P*< 0.001), and Model 3 (HR 2.99, 95% CI 1.89-4.72, *P* < 0.001) (**Table 2**).

Regarding the risk of cardiovascular mortality, the interaction *P*-values between key subgroup characteristics and NLR were all greater than 0.05, except for the CVD subgroup, indicating that the results still exhibit a high degree of consistency (**Figure 5B**).

### 3.4 The predictive capacity of NLR for mortality among participants with kidney stones

**Figure 7:**
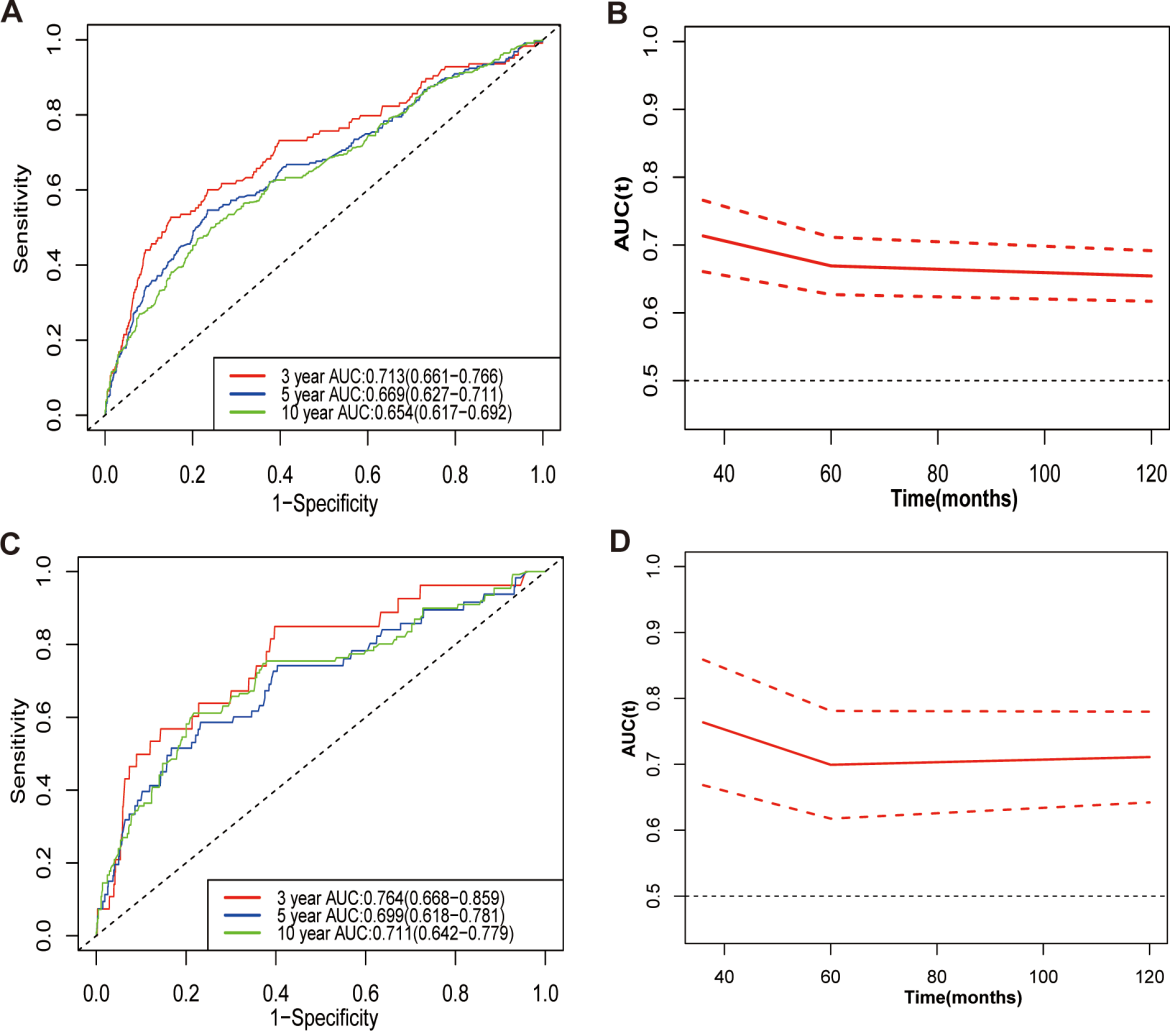
Time-dependent ROC curves for NLR: predicting 3, 5, and 10-year all-cause **(A, B)** and cardiovascular disease mortality **(C, D)**. AUC, area under curve.

**Figure 8:**
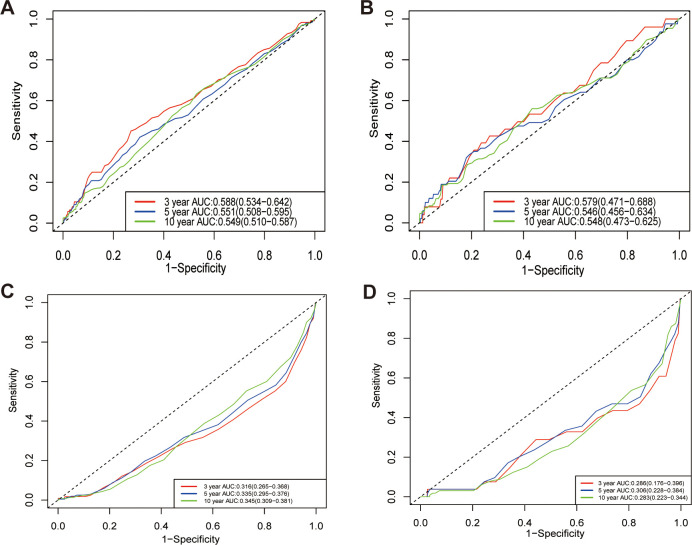
Time-dependent ROC curves for neutrophils and lymphocytes: all-cause **(A, B)** and cardiovascular mortality **(C, D)** predictions. **A** and **C** represent neutrophils; **B** and **D** represent lymphocytes. AUC, area under curve.

Time-dependent receiver operating characteristic (ROC) analysis, along with the calculation of the area under the curve (AUC), was employed to assess NLR to predict mortality risk. The AUC for NLR in predicting all-cause mortality at 3, 5, and 10 years was 0.713, 0.669, and 0.654, respectively (**Figure 7A**). Additionally, the AUC for NLR predicting cardiovascular mortality at 3, 5, and 10 years was 0.764, 0.699, and 0.711, respectively (**Figure 7B**). These results indicate that NLR has a high and consistent predictive ability for mortality in kidney stone patients over different time periods. Additionally, the predictive capabilities of lymphocytes and neutrophils independently were assessed for all-cause and cardiovascular mortality in participants with kidney stones, highlighting the advantages of NLR as a composite indicator. The findings indicated that NLR exhibited a greater predictive capacity for both all-cause and cardiovascular mortality than either neutrophils or lymphocytes alone, regardless of the time points evaluated (3, 5, or 10 years) (**Figure 8**).

**Figure 9:**
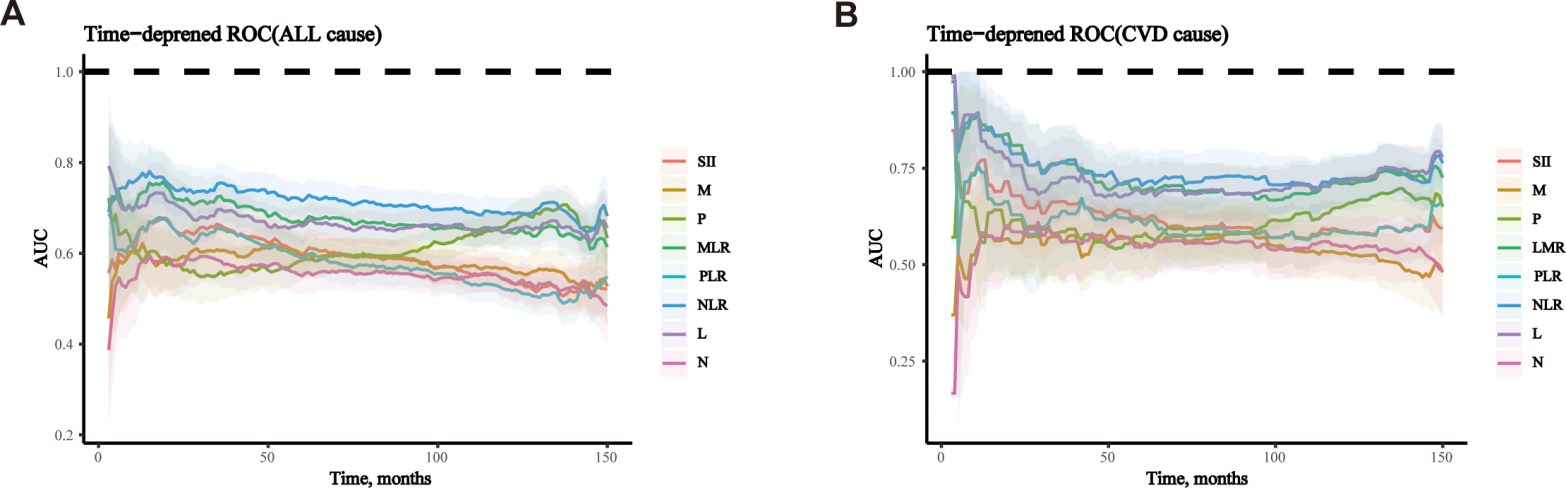
Time-dependent ROC curves of NLR and other inflammatory markers. **(A)** all-cause mortality **(B)** cardiovascular disease mortality. AUC, area under curve; NLR, neutrophil-to-lymphocyte ratio; SII, systemic immune-inflammation index; PLR, platelet-lymphocyte ratio; LMR, lymphocyte-monocyte ratio; M, monocytes; P, platelets; L, lymphocytes; N, neutrophils.

To validate the predictive performance of NLR, we compared its predictive ability with that of the systemic immune-inflammation index (SII), platelet-lymphocyte ratio, lymphocyte-monocyte ratio, as well as the counts of neutrophils, lymphocytes, platelets, and monocytes. We plotted the time-dependent ROC curves for all-cause and cardiovascular mortality, and the results showed that NLR had the largest (AUC) compared to several other inflammatory markers, as shown in **Figure 9**.

### 3.5 Mediation analysis of NLR in mortality among participants with kidney stones

**Figure 10:**
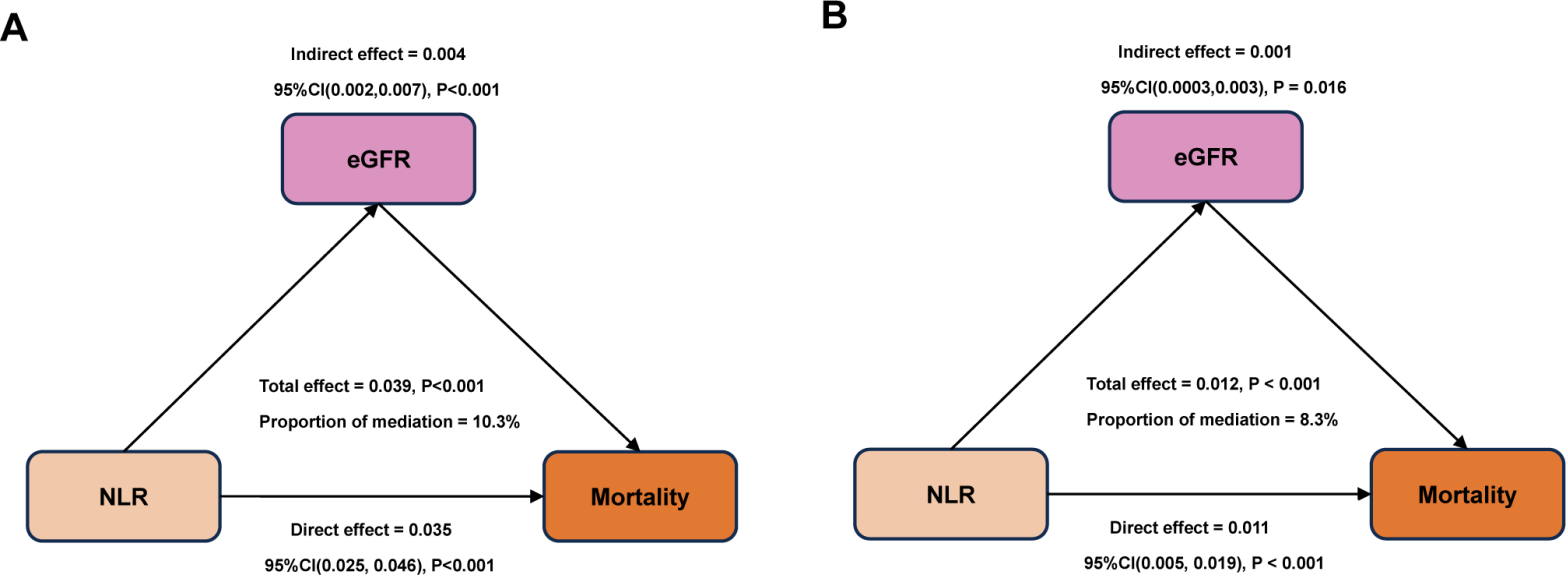
The mediating effect of eGFR on the relationship between NLR and mortality [**(A)**, all-cause death; **(B)**, cardiovascular death].

We also conducted a mediation effect analysis to evaluate the potential mediating effect of NLR on mortality and ultimately found that eGFR may serve as a significant mediating molecule. The results indicated that 10.3% of all-cause mortality and 8.3% of cardiovascular mortality were attributable to eGFR, representing a significant mediator in both instances (**Figure 10**).

### 3.6 Sensitivity analysis

To verify the reliability of the results, we also conducted a sensitivity analysis. Firstly, we made further adjustments for diet, commonly used medications, and health insurance, as shown in **Table 3**. Additionally, we excluded participants who died within 2 years of follow-up, as shown in **Table 4**.

**Table 3:** The relationships between NLR and mortality in participants with kidney stone.

Characteristics	Model 4 HR (95% CI)	P value	Model 5 HR (95% CI)	P value	Model 6 HR (95% CI)	P value
All-cause mortality						
NLR	1.15 (1.11, 1.20)	<0.001	1.15 (1.10, 1.20)	<0.001	1.15 (1.10, 1.20)	<0.001
NLR category						
Lower NLR	Ref.	Ref.	Ref.	Ref.	Ref.	Ref.
Higher NLR	2.35 (1.87, 2.97)	<0.001	2.36 (1.85, 3.01)	<0.001	2.37 (1.86, 3.02)	<0.001
Cardiovascular mortality						
NLR	1.18 (1.10, 1.28)	<0.001	1.17 (1.08, 1.27)	<0.001	1.17 (1.07, 1.27)	<0.001
NLR category						
Lower NLR	Ref.	Ref.	Ref.	Ref.	Ref.	Ref.
Higher NLR	3.01 (1.87, 4.83)	<0.001	3.01 (1.83, 4.93)	<0.001	2.97 (1.81, 4.87)	<0.001

Model 4, adjusted for age, sex, race, poverty income ratio, education level, smoking status, physical activity, body mass index, total cholesterol, high-density lipoprotein, estimated glomerular filtration rate, diabetes, hypertension, cardiovascular disease, cancer, and drugs(anti-diabetic drugs, anti-hypertensive drugs, anti-hyperlipidemic drugs); Model 5, adjusted for HEI-2015 on the basis of Model 4; Model 6, adjusted for health insurance on the basis of Model 5.

**Table 4:** The relationships between NLR and mortality in participants with kidney stone after exclusion of those who died within first 2 years of follow-up.

Characteristics	Model 1 HR (95% CI)	P value	Model 2 HR (95% CI)	P value	Model 3 HR (95% CI)	P value
All-cause mortality						
NLR	1.25 (1.19, 1.30)	<0.001	1.12 (1.07, 1.18)	<0.001	1.14 (1.08, 1.20)	<0.001
NLR category						
Lower NLR	Ref.		Ref.		Ref.	
Higher NLR	2.95 (2.25, 3.87)	<0.001	1.78 (1.35, 2.35)	<0.001	1.79 (1.35, 2.37)	<0.001
Cardiovascular mortality						
NLR	1.28 (1.19, 1.38)	<0.001	1.17 (1.08, 1.27)	0.001	1.19 (1.08, 1.31)	<0.001
NLR category						
Lower NLR	Ref.		Ref.		Ref.	
Higher NLR	4.11 (2.42, 7.00)	<0.001	2.64 (1.52, 4.58)	<0.001	2.54 (1.45, 4.46)	0.001

For All-caused-mortality.

Model 1, unadjusted; Model 2, adjusted for age, sex, race; Model 3, adjusted for age, sex, race, poverty income ratio, education level, smoking status, physical activity, body mass index, total cholesterol, high-density lipoprotein, estimated glomerular filtration rate, diabetes, hypertension, cardiovascular disease, and cancer.

For CVD-caused-mortality.

Model 1, unadjusted; Model 2, adjusted for age; Model 3, adjusted for age, estimated glomerular filtration rate, diabetes, cardiovascular disease.

## 4 Discussion

This study employed a nationwide large-sample analysis to examine the association between NLR and all-cause as well as cardiovascular mortality in patients with kidney stones. We analyzed relevant clinical data from 2,995 kidney stone participants in the NHANES 2007-2018 cycles and found that NLR was positively correlated with both all-cause mortality and cardiovascular mortality. A correlation was identified between NLR and both all-cause mortality and cardiovascular mortality in a cohort of 2,995 individuals with kidney stones, as evidenced by an examination of pertinent clinical data from the NHANES 2007-2018 cycles. When compared to neutrophils and lymphocytes alone, NLR demonstrated a greater capacity to predict mortality risk at 3, 5, and 10-year periods. Furthermore, mediation analysis revealed that eGFR acts as a mediator. Sensitivity analysis and stratified analysis confirmed the stability of the results.

NLR is constituted of neutrophils and lymphocytes, the data of which can be obtained via complete blood count (CBC). This makes it advantageous in terms of its low cost and ease of implementation. Research has shown that a higher neutrophil count indicates a persistent, non-specific inflammatory state in the body, while a lower lymphocyte count suggests a relative deficiency in immune status (28). NLR, as a composite marker, effectively reflects two opposing immune pathways, and its predictive ability is superior to that of parameters considering neutrophils or lymphocytes alone (29). In a previous study that also utilized the NHANES database, researchers included 21,106 participants and found that those with a higher NLR (>1.72) had an 18% increased risk of developing kidney stones and experiencing more episodes of stone passage (30). CRP is another common inflammatory marker, and higher levels of CRP are significantly associated with an increased prevalence of kidney stones (31, 32). SII is similarly a prevalent marker of inflammation, comprising the quantification of neutrophil, lymphocyte, and platelet counts. A previous study found that SII is positively correlated with the risk of kidney stones in American adults under the age of 50 (33). Although previous studies have reported the relationship between NLR and the risk of developing kidney stones, there is currently no research examining the impact of NLR on mortality rates among patients with kidney stones.

The ability of NLR to predict mortality risk in the general population has been confirmed (7). Furthermore, there is a notable correlation between NLR and an elevated risk of mortality in specific demographic groups, such as those with diabetes, hypertension, rheumatoid arthritis, chronic kidney disease, tumors, asthma and renal cell carcinoma (14, 34–39). Similar to these studies, our study also found that NLR is significantly correlated with the risk of mortality from all causes and cardiovascular disease in individuals with kidney stones.

This study is the first large-scale national study to confirm the prognostic impact of NLR on patients with kidney stones. NLR is associated with an increased risk of all-cause and cardiovascular mortality. The possible mechanisms are as follows. Firstly, NLR can affect renal function in patients with kidney stones; for example, NLR is associated with an increased risk of chronic kidney disease in patients with uric acid kidney stones (40). In this study, similar findings were observed, indicating that eGFR was significantly higher in the high NLR group compared to the low NLR group. Additionally, our mediation analysis demonstrated that eGFR functions as a mediating variable in the association between NLR and mortality risk. Secondly, the formation and progression of kidney stones is associated with inflammatory mechanisms. Neutrophils are related to the production of reactive oxygen species (ROS) (41). The production of ROS and the activation of inflammasomes are involved in the process of kidney stone formation (42). Exosomes can induce renal tubular cells to produce increased levels of IL-8, leading to the chemotactic migration and accumulation of neutrophils. This results in an increase in the fragility of exosomes, ultimately contributing to the formation of kidney stones (43). It has been confirmed in animal models that controlling inflammatory responses can prevent the recurrence of kidney stones (44). Finally, inflammation can also predict the occurrence of postoperative complications related to kidney stones. Percutaneous nephrolithotomy (PCNL) is the most common treatment for kidney stones due to its advantages of minimal trauma, shorter hospital stay, and quicker recovery. However, some complications still exist that can affect its prognosis, such as systemic inflammatory response syndrome (45). A clinical study utilizing machine learning found that a model incorporating NLR demonstrates high efficacy in predicting the incidence of postoperative systemic inflammatory response syndrome (46). In addition, regarding the risk of cardiovascular mortality, it was found that there was an interaction between NLR and CVD. The specific mechanism may be that the prevalence of CVD in the high NLR group is higher than that in the low NLR group, thereby increasing the risk of death from cardiovascular diseases. Therefore, NLR may influence prognosis and mortality risk by reducing renal function, increasing the recurrence of kidney stones, and lowering complications.

Our study has several advantages. At first, the use of a national multi-stage complex sampling strategy ensures the reliability of the results. Secondly, our study indicates that eGFR may play a mediating role in the association between NLR and mortality in individuals with kidney stones. Finally, multivariable adjustment and stratified analysis ensure the stability of the results.

However, this study has several limitations. Firstly, in this study, the diagnosis of kidney stones relied on participants’ questionnaires rather than more reliable imaging methods such as ultrasound or CT scans. This may lead to certain inaccuracies in the diagnosis of kidney stones. Some patients who actually had stones but were unaware of it or did not report it accurately might be missed, thereby affecting the reliability of the research results; Secondly, although we have adjusted many covariates, there may still be some important factors that have not been taken into account; Finally, the data used in this study is derived from kidney stone patients in the United States, therefore, future prospective studies from other countries and regions are needed to confirm the value of NLR in predicting the risk of mortality in patients with kidney stones.

## 5 Conclusion

Our study found that a high NLR is significantly and independently associated with an increased risk of mortality in patients with kidney stones. This may have important implications for the management of kidney stones; however, further research is still needed to explore the potential underlying mechanisms.

## Data Availability

The original contributions presented in the study are included in the article/supplementary material. Further inquiries can be directed to the corresponding author.
